# Angiotensin System Autoantibodies Correlate With Routine Prognostic Indicators for COVID-19 Severity

**DOI:** 10.3389/fmed.2022.840662

**Published:** 2022-03-09

**Authors:** Carmen M. Labandeira, Maria A. Pedrosa, Juan A. Suarez-Quintanilla, María Cortes-Ayaso, José Luis Labandeira-García, Ana I. Rodríguez-Pérez

**Affiliations:** ^1^Hospital Alvaro Cunqueiro, University Hospital Complex, Vigo, Spain; ^2^Research Center for Molecular Medicine and Chronic Diseases (CIMUS), Health Research Institute of Santiago de Compostela (IDIS), University of Santiago de Compostela, Santiago de Compostela, Spain; ^3^Primary Health-Care Unit Fontiñas, Health Research Institute of Santiago de Compostela (IDIS), University of Santiago de Compostela, Santiago de Compostela, Spain; ^4^Emergency Department, University Clinical Hospital of Santiago, Santiago de Compostela, Spain; ^5^Networking Research Center on Neurodegenerative Diseases (CIBERNED), Madrid, Spain

**Keywords:** ACE2 autoantibodies, angiotensin, COVID-19, hemoglobin, random forest algorithm, procalcitonin, AT1 autoantibodies, lactate dehydrogenase

## Abstract

**Objective:**

We previously showed that angiotensin type-1 receptor and ACE2 autoantibodies (AT1-AA, ACE2-AA) are associated with COVID-19 severity. Our aim is to find correlations of these autoantibodies with routine biochemical parameters that allow an initial classification of patients.

**Methods:**

In an initial cohort of 119 COVID-19 patients, serum AT1-AA and ACE2-AA concentrations were obtained within 24 h after diagnosis. In 50 patients with a complete set of routine biochemical parameters, clinical data and disease outcome information, a Random Forest algorithm was used to select prognostic indicators, and the Spearman coefficient was used to analyze correlations with AT1-AA, ACE2-AA.

**Results:**

Hemoglobin, lactate dehydrogenase and procalcitonin were selected. A decrease in one unit of hemoglobin, an increase in 0.25 units of procalcitonin, or an increase in 100 units of lactate dehydrogenase increased the severity of the disease by 35.27, 69.25, and 3.2%, respectively. Our binary logistic regression model had a predictive capability to differentiate between mild and moderate/severe disease of 84%, and between mild/moderate and severe disease of 76%. Furthermore, the selected parameters showed strong correlations with AT1-AA or ACE2-AA, particularly in men.

**Conclusion:**

Hemoglobin, lactate dehydrogenase and procalcitonin can be used for initial classification of COVID-19 patients in the admission day. Subsequent determination of more complex or late arrival biomarkers may provide further data on severity, mechanisms, and therapeutic options.

## Introduction

Severe Acute Respiratory Syndrome Coronavirus-2 (SARS-CoV-2) is a new strain of coronavirus, not previously identified in humans, responsible for the COVID-19 pandemic (1). Clinical manifestations and evolution of infected people are different depending on the prior characteristics of the patients. WHO Clinical Progression Scale (2) establishes five categories depending on the patient's state: uninfected (score 0), ambulatory mild disease (score 1–3); hospitalized moderate disease (score 4–5); hospitalized severe disease (score 6–9) and death (score 10). Most infected patients will develop mild to moderate illness characterized by fever, cough, and fatigue (3, 4). However, between 15.7 and 26.1% of patients will develop a severe manifestation of the illness characterized by severe pneumonia, organ failure and death (5). The magnitude of the COVID-19 pandemic and its devastating effects have been revealed by data presented on November 2, 2021, by the WHO that notified a total of 246.951.274 diagnosticated cases of COVID-19 in the world and a total of 5.004.855 deaths caused by SARS-CoV-2, quantified since January 27, 2020 (6). These alarming numbers could worsen, especially in developing countries, with the spread of the new variants that present different profiles of virus transmission, pathogenesis and vaccine efficacy (7–10).

Important advances in treating patients with severe disease have been developed (11), but treatments require expensive sanitary facilities like intensive care units (ICUs) that can provide oxygen, 24-h monitoring and care and assisted ventilation. ICU beds are limited in the sanitary centers and hospitals, especially in developing countries with high prevalence of COVID-19 disease (12). It is therefore essential to identify patients with high risk of severe illness to optimize correct distribution of these scarce health care resources to ensure proper treatment (13). Initial classification of patients may be done with routine laboratory tests that show results in a minimum time. After a first screening, the study of potential severe patients may be completed with more complex or late arrival biomarkers to provide further information on severity, mechanisms involved in severity and possible therapeutical strategies. In this study, we aimed to identify biochemical routine parameters used in clinical practice for initially prognosing risk of COVID-19 severity, using a machine-learning method (Random Forest). Furthermore, we studied their correlation with Renin Angiotensin System (RAS) autoantibodies, recently associated with severity of COVID-19 (14–16).

Angiotensin converting enzyme 2 (ACE2) is the SARS-CoV-2 virus entry receptor and it is also a major component of the RAS. The tissue RAS consists of two arms that counteract each other: a pro-inflammatory and pro-oxidative axis mainly formed by angiotensin II (Ang II) and its angiotensin type 1 (AT1) receptors, and an anti-inflammatory and anti-oxidative axis (17, 18). Since ACE2 transforms components of the pro-inflammatory RAS axis such as Ang II into components of the anti-inflammatory axis such as Ang 1–9 and particularly Ang 1–7, dysregulation of tissue RAS (by SARS-CoV-2 binding to ACE2) has been proposed as a major mechanism of progression of COVID-19 severity (19). Previous reports have shown that autoantibodies against AT1 receptors (AT1-AA; AT1 agonists) and for ACE2 (ACE2-AA, ACE2 antagonist) contribute to enhance the pro-inflammatory RAS axis (20), and in a previous study we observed significant association between AT1-AA, ACE2-AA and severity of COVID-19 outcome, suggesting that these autoantibodies could be used as an index of progression of COVID-19 toward severity (16). In the present work, we studied possible correlations between biochemical routine parameters and RAS autoantibodies. We first identified Procalcitonin (PCT); Hemoglobin (Hb) and lactate dehydrogenase (LDH) as predictors of severity using a machine learning method. Then, using the identified predictors, two models of logistic regression were used to predict COVID-19 severity, and the mathematical models were evaluated using area under the receiver operating characteristic curves (ROC), sensitivity and specificity metrics. Finally, we analyzed possible correlations between Hb, LDH, and PCT and the levels of autoantibodies against RAS (AT1-AA and ACE2-AA).

## Materials and Methods

### Study Design

This study was a part of a retrospective observational study where a total of 119 adult patients testing positive for SARS-CoV-2 RT-PCR were prospectively recruited from April 2020 to December 2020 at University Hospital Complex of Santiago de Compostela, University Hospital Complex of Santiago de Compostela Biobank and from Murcian Institute for Biosanitary Research Biobank (16). The eligibility criteria were as follows: patients who were diagnosed with COVID-19 using RT-PCR for SARS-CoV-2; to be older than 18 years old and to sign an informed consent (designed for this purpose). The study was approved by the Galician Drug Research Ethics Committee (CEIm-G), protocol 2020/212. The research was carried out in accordance with the principles of the Helsinki Declaration.

### Data Collection

Clinical data assessing demographics, comorbidities, symptoms, physical findings, disease stage, treatment, and laboratory tests results (including lymphocytes, Hb, D-dimer, LDH, C-reactive protein, creatinine, serum aminotransferases, ferritin, PCT and triglycerides) were collected from electronic medical records using a standardized data collection form. Only patients with complete usual routine biochemical parameters obtained within 24 h after diagnosis were included in the study to avoid biases in the statistical analysis (*n* = 50).

The main demographic and clinical characteristics of COVID-19 patients at the time point of data sampling are reported in Supplementary Table 1. Clinical outcome of patients was followed until medical discharge and, finally, patients were divided into three groups according to WHO Clinical Progression Scale (3): mild, moderate, or severe. Mild disease (scores 1–3; *n* = 16) patients were enrolled from the Emergency Room Department. Samples from moderate (scores 4–5; *n* = 20) and severe (scores 6–10; *n* = 14) hospitalized patients were obtained from the Emergency Room Department before hospitalization and from the University Hospital Complex of Santiago de Compostela Biobank and from Murcian Institute for Biosanitary Research Biobank. All patients were followed-up with electronic health information system until January 2021.

### Serum Samples and Anti-AT1 and Anti-ACE2 Autoantibody Measurements

Serum samples were obtained in the first 24 h after the diagnosis of COVID-19. After collection of the whole blood, the blood was allowed to clot by leaving it undisturbed at room temperature (30 min). Then the clot was removed by centrifugation at 1,500 × g for 20 min. Finally, resulting serum samples were stored at −80°C until analysis. Levels of AT1-AA and ACE2-AA were measured using two specific solid-phase, sandwich enzyme-linked immunosorbent assays (ELISAs) designed for their quantification (Catalog Number 12000 and 16000, respectively; Cell Trend; Luckenwalde, Germany). Determinations were performed strictly following the manufacturer's instructions. Absorbance was measured with an Infinite M200 multiwell plate reader (TECAN) at 450/620 nm. AT1-AA and ACE2-AA concentrations were determined using specific standard curves (4PL curve fit). Samples with values over the standard curve were diluted with assay buffer to get their absorbances within the standard curve. Positive and negative controls supplied by the manufacturer were assayed in parallel with the samples of interest. We also tested, as negative control, the sample buffer dilution without serum sample. Finally, we also tested samples in uncoated wells. Absorbances for negative controls resulted in values below the limit of detection of the standard curve.

### Statistical Methods

Data are expressed as mean ± standard deviation (SD). To test if the populations followed a normal distribution, Anderson-Darling test was used, and Fligner-Killeen test was used to check homoscedasticity. When data sets were normally distributed, two group comparisons were carried out by two tailed Student's *t*-test, with Welch correction if there is no equality of variances, and multiple comparisons by one-way ANOVA. Wilcoxon test for two group comparisons and Kruskal-Wallis test were used when data sets did not adjust to a normal distribution. Lymphocyte count (× 10^6^/L), Hb (g/dL), D-dimer (mg/L), LDH (U/L), c-reactive protein (mg/dL), PCT (ng/mL) and creatinine (mg/dL) were used to undergo random forest algorithm screening, and predictive models containing prognostic indicators for the disease severity were constructed with a combination of variables with lower OOB (out of bag) error. “VSURF” package in R, based on Random Forests, was used to select the most relevant features among the whole set of biochemical variables with default parameters, 2,000 tress and 10 variables randomly sampled as candidates at each split.

The selected variables were then used to build a Random Forest classifier with the “VSURF” package (21), and an ordinal logistic regression model, using the “MASS” package (22) and the “brant” package (23) in order to check the parallel regression assumption with a Brant test for the purpose of validating this ordinal logistic model. The predictive capability of these models was assessed following a leave-one-out cross validation strategy, and their accuracy in classifying patients according to mild, moderate and severe was also measured. Furthermore, binomial logistic regressions were adjusted with these predictors to assess the discriminant capability between (1) patients with mild and moderate or severe symptoms; and (2) patients with mild or moderate and severe symptoms. This binary classification was evaluated by using area under the receiver operating characteristic curve (ROC) and sensitivity and specificity metrics. Correlation coefficient (ρ) was used to study the correlation between biochemical routine parameters used in clinical practice and serum levels of AT1-AA and ACE2-AA. Associations between categorical variables were tested by using the chi-square test or the Fisher's exact test if expected value is <6 in a cell of the contingency table. *P* < 0.05 was considered significant for all the analyses. All the analyses were performed with R Software Version 4.0.3 (24).

## Results

### Prognostic Indicators Causing Critical Illness

#### Variable Selection

**Figure 1:**
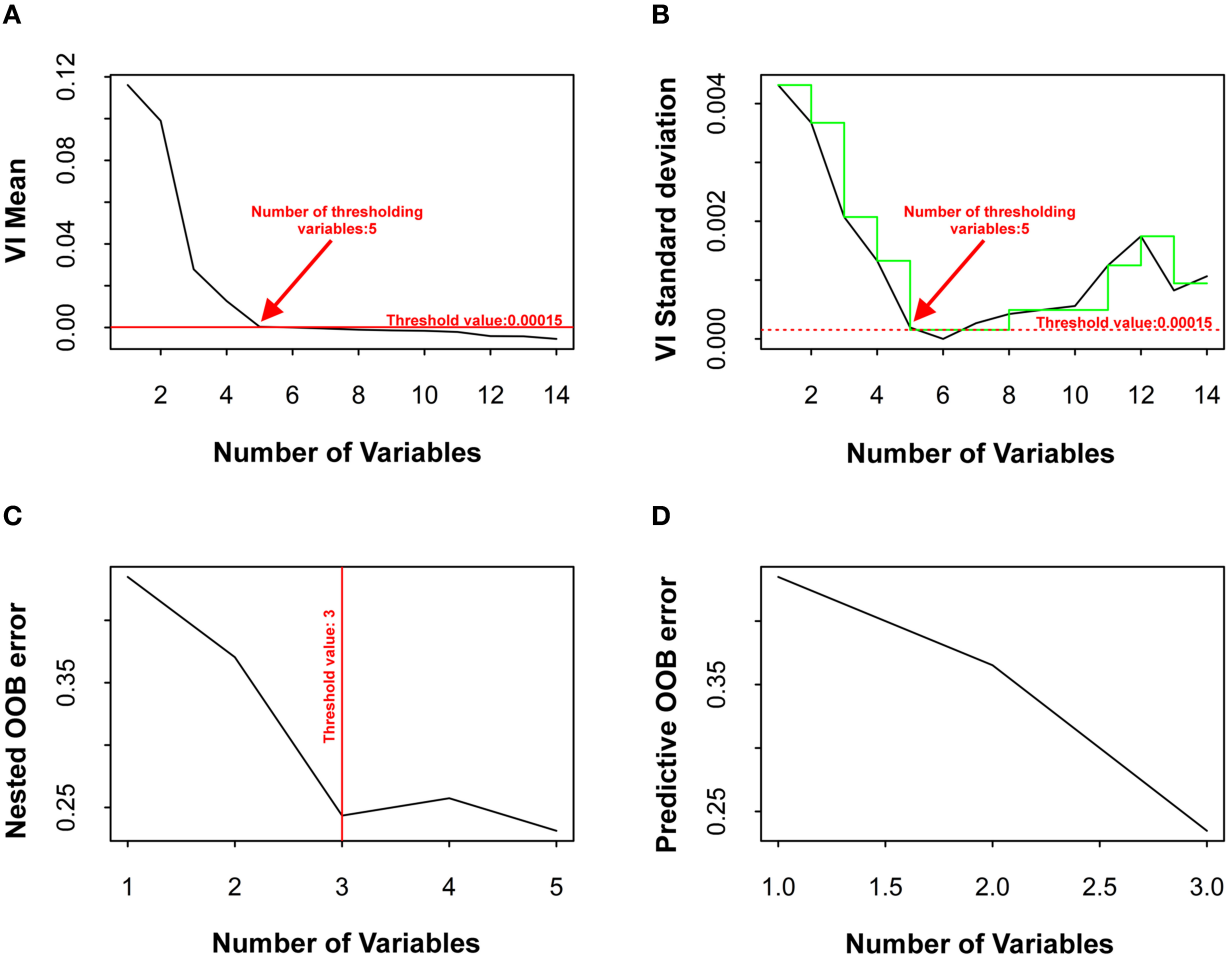
Steps of VSURF approach for variable selection: thresholding step **(A,B)**, to eliminate irrelevant variables from the dataset; interpretation step **(C)** to select all variables related to the response and prediction step **(D)** that refines the selection by eliminating redundancy in the set of variables. Black line in **(A)** represents the mean variable importance in decreasing order. The red horizontal line represents the value of the threshold in which there are no more relevant variables. This thresholding step selected five variables. Black curve in **(B)** represents the standard deviation of variable importance of the predictions given by a fitted CART tree. The dotted horizontal red line represents the minimum value of the standard deviation, i.e., value of the threshold. **(C)** Mean out-of-bag (OOB) error rate of embedded random forests models, from the one with only one variable as predictor, to the one with all variables kept after the previous step. The vertical red line indicates the retained model, composed by three variables. **(D)** Mean OOB error rate of embedded random forests models, being that variables added to the model in a stepwise manner here. The retained model is the final one, with three variables.

Three parameters were selected with the Random Forest algorithm in VSURF as final prognostic indicators: Hb, LDH and PCT (Figure 1).

#### Ordinal Logistic Regression Model

The calculated model with these three variables was:


$$\begin{array}{l} \log\left[\frac{P\left(Mild\;\geq Moderate\right)}{1-P\left(Mild\geq \;Moderate\right)}\right]=-6.1839\\ +\;0.0003235\left(LDH\right)-0.4349\left(Hb\right)+1.3290\left(PCT\right)\\ \log\left[\frac{P\left(Moderate\;\geq Severe\right)}{1-P\left(Moderate\geq \;Severe\right)}\right]=\;-3.9087\\ +\;0.0003235\left(LDH\right)-0.4349\left(Hb\right)+1.3290\left(PCT\right) \end{array}$$

This model shows that: an increase in one unit of LDH increases the odd (risk) of suffering a greater severity of the disease by exp (0.0003235) = 1.000324 (3.2% increase by 100 units, U/L). Given that OR = exp (−0.4349) = 0.6473, a decrease of one unit in Hb increases the odd of suffering a greater severity of the disease by 35.27% (g/dL). Given that OR = exp (1.329028) = 3.777, an increase of one unit in PCT increases the odd of suffering a greater severity by 277% (69.25% increase by 0.25 units, ng/ml).

Confusion matrix of the ordinal logistic regression model (Table 1) shows an accuracy of 62%.

**Table 1:** Confusion matrix of the ordinal logistic regression model for differentiating mild, moderate and severe patients.

**Severity**	**Mild**	**Moderate**	**Severe**	**Total real**
Mild	12	4	0	16
Moderate	4	12	4	20
Severe	2	5	7	14

*The model predicts 12 mild severity patients vs. 16 real mild severity patients; 12 moderate patients vs. 20 real and 7 severe patients vs. 14 real, showing an accuracy of 62%*.

#### Random Forest Classifier

With the selected variables, a Random Forest model was adjusted with default parameters using the VSURF package in R. The accuracy of this classifier was evaluated by using a leave-one-out cross validation strategy, and the results of the predictions were represented in the following confusion matrix (Table 2). The accuracy for this classifier was 70%.

**Table 2:** Confusion matrix of the default parameters.

**Severity**	**Mild**	**Moderate**	**Severe**	**Total real**
Mild	14	2	0	16
Moderate	2	14	4	20
Severe	2	5	7	14

*Leave-one-out cross validation strategy shows that 14 mild patients were classified as mild vs. 16 real, 14 moderate patients vs. 20 real and 7 severe patients vs. 14 real, showing an accuracy of 70%*.

#### Binomial Logistic Models

In order to further analyze the reliability of the model, binomial logistic regressions were applied to construct a joint model that contains the selected prognostic indicators. Patients were discriminated into two groups according to distinguish those that will present mild severity from those that will present moderate or severe disease and those with mild or moderate disease from those with severe disease.

The logistic regression model obtained for differentiating mild from moderate or severe patients was:


$$\begin{array}{l} \log\left[\frac{P\left(Moderate/Severe\right)}{1-P\left(Moderate/Severe\right)}\right]=0.7925+0.0169\left(LDH\right)\\ \;-0.3461\left(Hb\right)-1.7402\left(PCT\right) \end{array}$$

**Figure 2:**
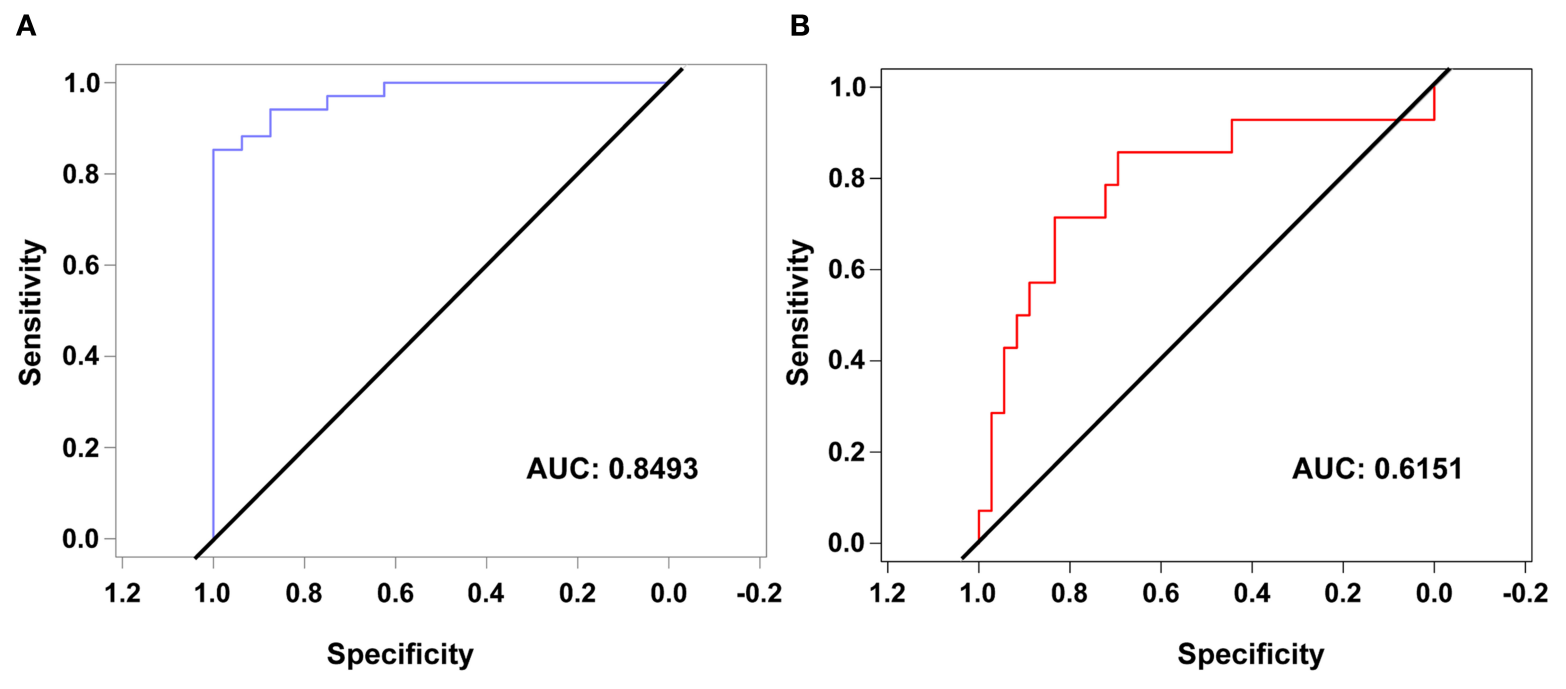
Receiver operating characteristic (ROC) curves of the obtained ordinal logistic model for differentiating mild from moderate or severe patients **(A)** and for differentiating severe patients from mild or moderate patients **(B)**.

To evaluate the predictive capability of the model a confusion matrix was used (Table 3). 84% of predictions were correctly classified. The area under the ROC curve was 0.8493 with a confidence interval of (0.7433–0.9552), a sensitivity of 0.824 and a specificity of 0.875 (Figure 2A).

**Table 3:** Confusion matrix of the ordinal logistic regression model for differentiating mild from moderate or severe patients.

**Severity**	**Mild**	**Moderate/severe**	**Total real**
Mild	14	2	16
Moderate/severe	6	28	34

*The model predicts 14 mild patients vs. 16 real mild patients and 28 moderate/severe patients vs. 34 real, showing an accuracy of 84%*.

The logistic regression model obtained for differentiating mild or moderate severity from severe patients was:


$$\begin{array}{l} \log\left[\frac{P\left(Severe\right)}{1-P\left(Severe\right)}\right]=2.5635-0.0001\left(LDH\right)-0.3126\left(Hb\right)\\ \;+\;1.8865\left(PCT\right) \end{array}$$

Again, to evaluate the predictive capacity of the model a confusion matrix was used (Table 4). 76% of predictions were correctly classified. The area under the ROC curve was 0.6151 with a confidence interval of (0.50–0.7436), a sensitivity of 0.286 and a specificity of 0.944 (Figure 2B).

**Table 4:** Confusion matrix of the ordinal logistic regression model for differentiating severe patients from mild or moderate patients.

**Severity**	**Mild/moderate**	**Severe**	**Total real**
Mild/moderate	34	2	36
Severe	10	4	14

*The model predicts 34 mild/moderate patients vs. 36 real mild/moderate patients and 4 severe patients vs. 14 real, showing an accuracy of 76%*.

### Correlations Between Hb, PCT and LDH and AT1-AA and ACE2-AA Serum Levels

Strong significant negative correlation between serum levels of AT1-AA and Hb was observed (ρ = −0.568, *p* < 0.0001) in COVID-19 patients. Then, we decided to investigate this correlation by gender. Interestingly, it was stronger for men (ρ = −0.795, *p* < 0.0001) but lower in women (ρ = −0.254), where the significance disappeared (*p* = 0.220). We also observed an important significant positive correlation between AT1-AA and LDH serum levels in COVID-19 patients (ρ = 0.436; *p* = 0.002) that was stronger for men (ρ = 0.528; *p* = 0.008) and lower for women (ρ = 0.404; *p* = 0.045). A significant and positive correlation between AT1-AA and PCT was only found in men (ρ = 0.511; *p* = 0.011).

Strong significant negative correlations between serum levels of ACE2-AA and Hb were observed in total COVID-19 patient's (ρ = −0.702; *p* < 0.0001); male (ρ = −0.782; *p* < 0.0001) and female patients (ρ = −0.658; *p* = 0.0003). Correlations between ACE2-AA and LDH were strong in total patient's population (ρ = 0.519; *p* = 0.0001), men (ρ = 0.460, *p* = 0.0237) and women (ρ = 0.553, *p* = 0.0034). Finally, significant positive correlation was observed between ACE2-AA and PCT in COVID-19 patients (ρ = 0.313; *p* = 0.027) and men (ρ = 0.409; *p* = 0.047) but the correlation disappeared in women (ρ = 0.0645; *p* = 0.7543). Summary of the Spearman correlations is shown is Table 5.

**Table 5:** Summary of the Spearman correlation coefficients (ρ), *p*-values and confidence interval (CI) for the correlations between autoantibodies and the default biochemical parameters.

**Correlations**		**ρ**	***P***	**95% CI**
AT1-AA/Hb	Total	−0.568	<0.0001	95% CI = (−0.736, −0.333)
	Men	−0.795	<0.0001	95% CI = (−0.910, −0.568)
	Women	−0.254	0.220	95% CI = (−0.598, 0.169)
ACE2-AA/Hb	Total	−0.702	<0.0001	95% CI = (−0.823, −0.520)
	Men	−0.782	<0.0001	95% CI = (−0.903, −0.543)
	Women	−0.658	0.0003	95% CI = (−0.837, −0.352)
AT1-AA/LDH	Total	0.436	0.002	95% CI = (0.169, 0.644)
	Men	0.528	0.008	95% CI = (0.146, 0.773)
	Women	0.404	0.045	95% CI = (−0.002, 0.695)
ACE2-AA/LDH	Total	0.519	0.0001	95% CI = (0.274, 0.701)
	Men	0.46	0.0237	95% CI = (0.057, 0.734)
	Women	0.553	0.0034	95% CI = (0.200, 0.779)
AT1-AA/PCT	Total	0.149	0.306	95% CI = (−0.146, 0.420)
	Men	0.511	0.011	95% CI = (0.123, 0.763)
	Women	0.236	0.257	95% CI = (−0.188, 0.585)
ACE2-AA/PCT	Total	0.313	0.027	95% CI = (0.029, 0.550)
	Men	0.409	0.047	95% CI = (−0.006, 0.704)
	Women	0.0645	0.7543	95% CI = (−0.342, 0.451)

## Discussion

The present study was designed to know whether dysregulation of AT1 and ACE2 autoantibodies, which were associated with COVID-19 severity in several recent studies, may correlate with more simple parameters for initial classification of patients. This is still necessary in the management of the disease at the present time, when new and more lethal or infective variants of the virus are emerging worldwide. Our results show that three variables (Hb, LDH and PCT) measured at the time of hospital admission, correlate with AT1-AA and ACE2-AA, and provide a reference for predicting the severity of the disease. Further research on the correlation of AT1-AA and ACE2-AA with other autoantibodies and biological parameters that have been related to COVID-19, would contribute to confirmation of the accuracy of quick biochemical parameters, clarification of mechanisms involved in disease severity, and to design better therapeutical responses.

The classificatory capacity of the ordinal regression and the random forest exceeds 60%, a good percentage considering that there are three categories. The ordinal logistic regression model suggests that higher concentrations of Hb act as a “protective” factor against severe forms of the disease (severity and Hb are inversely related) and higher concentrations of LDH and PCT are associated with greater severity of the disease (severity and LDH and PCT are directly related). Interestingly, using conventional binary logistic regression, the ability to differentiate between mild and moderate/severe disease was very good with a predictive capacity of 84%. To distinguish between mild/moderate and severe disease the predictive capacity was 76%.

Our ordinal logistic regression model shows that the decrease in one unit of Hb increases the odd of suffering a greater disease severity by 35.27%. Recent studies also observed a strong association between low levels of Hb and severe progression of COVID-19 (25, 26). Several pathophysiological mechanisms could justify this association. Hb is the oxygen carrier and when its concentration decreases, oxygen transport to different organs is compromised, causing hypoxia that may result in the development of multiple organ dysfunction, especially respiratory organ dysfunction (27). This multiple organ dysfunction will contribute to the occurrence of severe and irreversible effects in COVID-19 patients. Interestingly, it was also suggested that SARS-CoV-2 can interact with erythrocyte Hb molecules through ACE2, CD147 and CD26 membrane receptors. This interaction may promote the virus attack to the heme on the 1-beta chain of Hb resulting in hemolysis (28). In addition, SARS-CoV-2 may mimic the action of hepcidin, increasing tissue and circulating ferritin, which promotes serum iron deficiency and lack of Hb. The outcome of these processes is hyperferritinemia and ferroptosis, which in turns generates high oxidative stress and lipoperoxidation promoting inflammatory/immune over-response (cytokine storm) (28). Furthermore, recent results obtained by (29) suggest that elevated IL-6, an important component of the cytokine storm, could result in progressive anemia (29). Therefore, a positive feedback mechanism may exist between COVID-19 and low Hb levels that amplifies the risk of severe COVID-19 and anemia outcomes. Consistent with the present results, a recent study shows that COVID-19 patients with anemia had an 8.2 times greater possibility of developing severe pneumonia compared with COVID-19 patients without anemia (30).

Our ordinal logistic regression model shows that an increase of 0.25 units in PCT increases the odd of suffering a greater severity by 69.25%. This finding is consistent with other recent studies that also observed a positive association between elevated PTC and COVID-19 severity (31, 32). PCT is the precursor peptide of the calcitonin. In healthy individuals, serum levels of PCT are <0.05 ng/mL, but levels are raised in response to a pro-inflammatory stimulus especially of bacterial origin. Furthermore, synthesis and secretion of PCT may also be induced indirectly via pro-inflammatory cytokines, such as tumor necrosis factor-α (TNF-α) and IL-6 (33). Therefore, two explanations may justify the increased PCT levels associated to severe COVID-19 outcome: first, the high serum levels of cytokines like IL-6 and TNF-α from “cytokine storm” that may promote the increase in PCT levels; second, the presence of a bacterial co-infection.

LDH is an enzyme that catalyzes the conversion of lactate to pyruvate. It is expressed in almost all body cells with highest levels in heart, liver, lungs, muscles, kidneys and blood cells. Because LDH is released during tissue damage, it is a marker of common injuries and disease. Our ordinal logistic regression model shows that the increase in 100 units of LDH increases the odd of suffering a greater severity by 3.2%. This is according to other studies reporting that high levels of LDH could be a severity biomarker in COVID-19 (34, 35).

In a recent work, we showed that autoantibodies against AT1 receptors (AT1-AA; AT1 receptor agonists) and for ACE2 (ACE2-AA, ACE2 antagonists) are associated with severity of COVID-19 outcome and could be used as an index of probable progression of COVID-19 toward severity (16), which was also observed in other recent studies (14, 15). In the present work, we show a correlation with routine biochemical parameters determined at hospital admission. A possible explanation for this correlation may be that AT1-AA act as agonists of AT1 receptors, which triggers pro-inflammatory actions mediated by the release of interleukins that, in turn, would be involved in the above-mentioned decrease in Hb levels and in the synthesis and release of PCT and LDH. Little is currently known about the role of ACE2-AA, which have been related to increased levels of soluble ACE2 (36). We suggest that binding of SARS-CoV-2 to the cellular membrane causes a decrease in transmembrane ACE2 and ACE2 shedding, leading to: (i) the increase in levels of Ang II available to interact with AT1 receptors, which would promote cytokine release that induces the above-mentioned decrease in Hb and increase in PCT and LDH levels; (ii) the increase in soluble/circulating ACE2 that may induce ACE2-AA, which may further inhibit both soluble and transmembrane ACE2 activities.

The gender differences in above-mentioned correlations between selected routine parameters and autoantibodies are more difficult to explain. One possible explanation could be gender differences found in several hematological and biochemical parameters in COVID-19 patient's, including not only well-known sex-specific parameters (such as Hb and hematocrit), but also PCT and LDH, which were substantially increased in men (37). In our study, the male cohort showed the most significant and strongest correlations for PCT/AT1-AA, PCT/ACE2-AA, LDH/AT1-AA and LDH/ACE2-AA. However, these correlations disappeared in the women cohort for PCT/AT1-AA, PCT/ACE2-AA, remaining strong and significant in the case of LDH/AT1-AA and LDH/ACE2-AA.

Our study has some limitations. A first limitation is the relatively low sample size. Severity groups were not well-balanced, as most of the patients belong to the moderate group. Another consideration is that incidence of COVID-19 may be different in different countries where new virus mutations are emerging. This fact could limit the extrapolation of results. Finally, this study involved only two centers (Hospital of Santiago de Compostela and Murcian Institute for Biosanitary Research Biobank), which may difficult the generalization of the obtained results to other settings and healthcare systems. Considering the potential of the obtained results, we think that the present model may be improved by implementing a multi-center study including a larger number of patients affected with new different viral variants.

## Data Availability Statement

The original contributions presented in the study are included in the article/Supplementary Material, further inquiries can be directed to the corresponding author/s.

## Ethics Statement

The studies involving human participants were reviewed and approved by Galician Drug Research Ethics Committee (CEIm-G), protocol 2020/212. The patients/participants provided their written informed consent to participate in this study.

## Author Contributions

AR-P, JL-G, and CL: conceptualization, methodology, formal analysis, writing-original, draft preparation, writing-review and editing, and supervision. JL-G and AR-P: project administration and funding acquisition. CL, MC-A, and JS-Q: subject selection, collecting samples, and clinical data. MP: AA measurements and analyses. All authors have read and agreed to the published version of the manuscript.

## Funding

This research was funded by Axencia Galega de Innovación, grant number IN845D 2020/20, Spanish Ministry of Economy and Competitiveness, grant number RTI2018-098830-B-I00, Spanish Ministry of Health, grant numbers PI20/00345 and RD16/0011/0016, Galician Government (XUGA) grant numbers ED431C 2018/10 and ED431G/05, and FEDER (Regional European Development Fund).

## Conflict of Interest

The authors declare that the research was conducted in the absence of any commercial or financial relationships that could be construed as a potential conflict of interest.

## Publisher's Note

All claims expressed in this article are solely those of the authors and do not necessarily represent those of their affiliated organizations, or those of the publisher, the editors and the reviewers. Any product that may be evaluated in this article, or claim that may be made by its manufacturer, is not guaranteed or endorsed by the publisher.
