# Supplementary material for: Angiotensin System Autoantibodies Correlate With Routine Prognostic Indicators for COVID-19 Severity
Source: Front Med (Lausanne). 2022 Mar 9;9:840662. doi: 10.3389/fmed.2022.840662 (PMC8959920; doi:10.3389/fmed.2022.840662)
Supplement: Supplementary file 1 [file Table_1.docx]

Supplementary Material

Angiotensin system autoantibodies correlate with routine prognostic indicators for COVID-19 severity

Carmen M. Labandeira^1,2^, Maria A. Pedrosa^2^, Juan A. Suarez-Quintanilla^4^, María Cortes-Ayaso^5^, José Luis Labandeira-García^2,3 *,†^ and Ana I Rodríguez-Pérez^2,3 *,†^

^1^ Hospital Alvaro Cunqueiro, University Hospital Complex, Vigo, Spain.

^2^ Research Center for Molecular Medicine and Chronic Diseases (CIMUS), IDIS, University of Santiago de Compostela, Santiago de Compostela, Spain.

^3^ Networking Research Center on Neurodegenerative Diseases (CIBERNED), Spain

^4^ Primary Health-Care Unit Fontiñas, IDIS, University of Santiago de Compostela, Santiago de Compo-stela, Spain.

^5^ Emergency Department, University Clinical Hospital of Santiago, Santiago de Compostela, Spain.

^†^ These authors have contributed equally to this work and share last authorship

*** Correspondence:**José Luis Labandeira-García: [joseluis.labandeira@usc.es](mailto:joseluis.labandeira@usc.es); Ana I Rodríguez-Pérez^:^ anai.rodriguez@usc.es

# Supplementary Table 1

|  | **Mild** | **Moderate** | **Severe** | **Total** | **P-value** |
| --- | --- | --- | --- | --- | --- |
| **ACE2-AA (U/mL)** | | | | | < 0.001 (1) |
| **N** | 16 | 20 | 14 | 50 |  |
| **Mean (SD)** | 4.600 (3.972) | 26.109 (27.628) | 30.856 (31.180) | 20.555 (26.168) |  |
| **Median** | 2.856 | 18.221 | 19.597 | 10.207 |  |
| **IQR** | 3.354 | 28.135 | 36.143 | 19.116 |  |
| **Q1, Q3** | 1.990, 5.344 | 5.994, 34.129 | 8.719, 44.862 | 4.049, 23.166 |  |
| **Range** | 1.125 - 14.648 | 0.246 - 113.685 | 0.599 - 93.113 | 0.246 - 113.685 |  |
| **AT1-AA (U/mL)** | | | | | 0.008 (2) |
| **N** | 15 | 20 | 14 | 49 |  |
| **Mean (SD)** | 5.949 (3.851) | 8.705 (1.980) | 8.697 (3.571) | 7.859 (3.312) |  |
| **Median** | 4.452 | 8.596 | 8.705 | 8.207 |  |
| **IQR** | 1.026 | 2.420 | 3.277 | 4.875 |  |
| **Q1, Q3** | 4.008, 5.064 | 7.500, 9.920 | 6.662,9.338 | 4.974, 9.848 |  |
| **Range** | 2.694 – 16.000 | 4.356 - 12.733 | 1.378 - 17.199 | 1.378 - 17.199 |  |
| **Age (Years)** | | | | | 0.002 (3) |
| **N** | 16 | 20 | 14 | 50 |  |
| **Mean (SD)** | 48.625 (12.717) | 59.900 (13.151) | 67.071 (14.339) | 58.300 (14.990) |  |
| **Median** | 49.5 | 60 | 65.5 | 58 |  |
| **IQR** | 17.5 | 18.75 | 15.5 | 21.25 |  |
| **Q1, Q3** | 40.5 58.0 | 50.5- 69.3 | 60.0. 75.5 | 47.5- 68.8 |  |
| **Range** | 20.0 - 70.0 | 41.0 - 84.0 | 41.0 - 93.0 | 20.0 - 93.0 |  |
| **Gender** | | | | | 0.322 (4) |
| **N** | 16 | 20 | 14 | 50 |  |
| **Men** | 6 (37.5%) | 9 (45.0%) | 9 (64.3%) | 24 (48.0%) |  |
| **Female** | 10 (62.5%) | 11 (55.0%) | 5 (35.7%) | 26 (52.0%) |  |
| **Obesity** | | | | | 0.352 (5) |
| **N** | 10 | 12 | 10 | 32 |  |
| **No** | 2 (20.0%) | 6 (50.0%) | 4 (40.0%) | 12 (37.5%) |  |
| **Yes** | 8 (80.0%) | 6 (50.0%) | 6 (60.0%) | 20 (62.5%) |  |
| **Hypertension** | | | | | 0.291 (4) |
| **N** | 16 | 20 | 14 | 50 |  |
| **Yes** | 3 (18.8%) | 8 (40.0%) | 6 (42.9%) | 17 (34.0%) |  |
| **No** | 13 (81.2%) | 12 (60.0%) | 8 (57.1%) | 33 (66.0%) |  |
| **Diabetes Mellitus** | | | | | 0.454 (5) |
| **N** | 16 | 20 | 14 | 50 |  |
| **Yes** | 1 (6.2%) | 3 (15.0%) | 3 (21.4%) | 7 (14.0%) |  |
| **No** | 15 (93.8%) | 17 (85.0%) | 11 (78.6%) | 43 (86.0%) |  |
| **Dyslipidemia** | | | | | 0.091 (4) |
| **N** | 16 | 20 | 14 | 50 |  |
| **Yes** | 3 (18.8%) | 7 (35.0%) | 8 (57.1%) | 18 (36.0%) |  |
| **No** | 13 (81.2%) | 13 (65.0%) | 6 (42.9%) | 32 (64.0%) |  |
| **Cardiovascular disease** | | | | | 0.616 (5) |
| **N** | 16 | 20 | 14 | 50 |  |
| **Yes** | 0 (0.0%) | 2 (10.0%) | 1 (7.1%) | 3 (6.0%) |  |
| **No** | 16 (100.0%) | 18 (90.0%) | 13 (92.9%) | 47 (94.0%) |  |
| **Chronic Respiratory Disease** | | | | | 0.596 (5) |
| **N** | 16 | 20 | 13 | 49 |  |
| **Yes** | 2 (12.5%) | 1 (5.0%) | 2 (15.4%) | 5 (10.2%) |  |
| **No** | 14 (87.5%) | 19 (95.0%) | 11 (84.6%) | 44 (89.8%) |  |
| **Nephropathy** |  |  |  |  | 0.470 (6) |
| **N** | 16 | 20 | 14 | 50 |  |
| **No nephropathy** | 16(100%) | 20(100%) | 14(100%) | 50(100%) |  |
| **Hemoblogin (g/dL)** | | | | | < 0.001 (7) |
| **N** | 16 | 20 | 14 | 50 |  |
| **Mean (SD)** | 14.800 (1.084) | 12.595 (2.124) | 11.229 (2.481) | 12.918 (2.399) |  |
| **Median** | 14.9 | 12.85 | 10.6 | 13.3 |  |
| **IQR** | 1.575 | 2.3 | 3.1 | 3.575 |  |
| **Q1, Q3** | 14.025, 15.600 | 11.400, 13.700 | 9.425, 12.525 | 11.275, 14.850 |  |
| **Range** | 13.300 - 16.600 | 7.700 - 15.900 | 8.100 - 17.300 | 7.700 - 17.300 |  |
| **Lymphocytes (x10^6^/L)** | | | | | 0.681 (8) |
| **N** | 12 | 20 | 14 | 46 |  |
| **Mean (SD)** | 1297.500 (453.895) | 1360.000 (513.092) | 1344.286 (1105.202) | 1338.913 (717.670) |  |
| **Median** | 1205 | 1240 | 1260 | 1255 |  |
| **IQR** | 405 | 822.5 | 795 | 785 |  |
| **Q1, Q3** | 982.500, 1387.500 | 1017.500, 1840.000 | 635.000, 1430.000 | 857.500, 1642.500 |  |
| **Range** | 750.000 - 2290.000 | 590.000 - 2110.000 | 410.000 - 4760.000 | 410.000 - 4760.000 |  |
| **D-Dimer (mg/L)** | | | | | 0.002 (9) |
| **N** | 15 | 17 | 13 | 45 |  |
| **Mean (SD)** | 662.267 (381.779) | 884.824 (885.649) | 3001.231 (3673.175) | 1422.044 (2248.647) |  |
| **Median** | 620 | 555 | 1451 | 666 |  |
| **IQR** | 511 | 376 | 2532 | 837 |  |
| **Q1, Q3** | 359.000, 870.000 | 444.000, 820.000 | 942.000, 3474.000 | 460.000, 1297.000 |  |
| **Range** | 215.000 - 1509.000 | 180.000 - 2992.000 | 489.000 - 13847.000 | 180.000 - 13847.000 |  |
| **Lactate Dehydrogenase (U/L)** | | | | | < 0.001 (1) |
| **N** | 16 | 20 | 14 | 50 |  |
| **Mean (SD)** | 192.312 (45.917) | 529.600 (769.415) | 476.571 (173.002) | 406.820 (510.592) |  |
| **Median** | 177.5 | 350 | 471 | 312.5 |  |
| **IQR** | 52.5 | 143.5 | 217.5 | 247.75 |  |
| **Q1, Q3** | 163.000, 215.500 | 291.750, 435.250 | 379.500, 597.000 | 190.500, 438.250 |  |
| **Range** | 133.000 - 320.000 | 189.000 - 3770.000 | 166.000 - 789.000 | 133.000 - 3770.000 |  |
| **C-Reactive Protein (mg/dL)** | | | | | 0.006 (10) |
| **N** | 16 | 20 | 14 | 50 |  |
| **Mean (SD)** | 3.120 (5.741) | 2614.177 (4820.556) | 3997.304 (5598.254) | 2165.914 (4460.167) |  |
| **Median** | 1.3 | 2.6 | 1829 | 2.615 |  |
| **IQR** | 1.745 | 2382.264 | 5075.16 | 2127.532 |  |
| **Q1, Q3** | 0.335. 2.080 | 0.236. 2382.5 | 5.340. 5080.500 | 0.469, 2128.0 |  |
| **Range** | 0.050 - 21.430 | 0.015 - 15859.0 | 0.016 - 16649.0 | 0.015 - 16649.0 |  |
| **Procalcitonin (ng/mL)** | | | | | 0.003 (10) |
| **N** | 16 | 20 | 14 | 50 |  |
| **Mean (SD)** | 0.068 (0.053) | 0.118 (0.205) | 0.588 (0.970) | 0.234 (0.563) |  |
| **Median** | 0.05 | 0.05 | 0.16 | 0.07 |  |
| **IQR** | 0.042 | 0.073 | 0.48 | 0.095 |  |
| **Q1, Q3** | 0.037. 0.080 | 0.037. 0.110 | 0.070. 0.550 | 0.040. 0.135 |  |
| **Range** | 0.020 - 0.220 | 0.020 - 0.950 | 0.050 - 3.300 | 0.020 - 3.300 |  |
| **Creatinine (mg/dL)** | | | | | 0.979 (11) |
| **N** | 16 | 20 | 14 | 50 |  |
| **Mean (SD)** | 0.751 (0.159) | 0.790 (0.249) | 0.820 (0.320) | 0.786 (0.244) |  |
| **Median** | 0.74 | 0.68 | 0.78 | 0.74 |  |
| **IQR** | 0.123 | 0.365 | 0.288 | 0.243 |  |
| **Q1, Q3** | 0.703. 0.825 | 0.610. 0.975 | 0.585. 0.873 | 0.635. 0.878 |  |
| **Range** | 0.360 - 1.030 | 0.480 - 1.350 | 0.500 - 1.680 | 0.360 - 1.680 |  |

**Supplementary Table 1. Main clinical features of patients and association with severity (p<0.05).** ACE2-AA: ACE2 autoantibodies; AT1-AA: Autoantibodies for AT1 receptors; IQR: Interquartile range; SD: Standard deviation. (1) Kruskal-Wallis rank sum test , AD test= 0 , FK test= 0.001; (2) Kruskal-Wallis rank sum test , AD test= 0 , FK test= 0.519; (3) Linear Model ANOVA , AD test= 0.71 , FK test= 0.935; (4) Pearson’s Chi-squared test; (5) Fisher’s Exact Test for Count Data; (6) Chi-squared test for given probabilities; (7) Linear Model ANOVA , AD test= 0.508 , FK test= 0.058; (8) Kruskal-Wallis rank sum test , AD test= 0.001 , FK test= 0.447; (9) Kruskal-Wallis rank sum test , AD test= 0 , FK test= 0.006; (10) Kruskal-Wallis rank sum test , AD test= 0 , FK test= 0; (11) Kruskal-Wallis rank sum test , AD test= 0.041 , FK test= 0.183. 1. Missing data occurred at random. Statistical analysis was performed with the available data and imputed data were not used. Mortality rate: 4 deaths/50 patients = 0.2 for 9 months.
